# The Oxytocin-Oxytocin Receptor System and Its Antagonists as Tocolytic Agents

**DOI:** 10.1155/2011/350546

**Published:** 2011-12-06

**Authors:** Nikolaos Vrachnis, Fotodotis M. Malamas, Stavros Sifakis, Efthymios Deligeoroglou, Zoe Iliodromiti

**Affiliations:** ^1^2nd Department of Obstetrics and Gynecology, Aretaieio Hospital, University of Athens Medical School, 11526 Athens, Greece; ^2^1st Department of Obstetrics and Gynecology, Alexandra Hospital, University of Athens Medical School, 11526 Athens, Greece; ^3^Department of Obstetrics and Gynaecology, University Hospital of Heraklion, 71110 Heraklion, Crete, Greece

## Abstract

Oxytocin, a hormone involved in numerous physiologic processes, plays a central role in the mechanisms of parturition and lactation. It acts through its receptor, which belongs to the G-protein-coupled receptor superfamily, while Gq/phospholipase C (PLC)/inositol 1,4,5-triphosphate (InsP3) is the main pathway via which it exerts its action in the myometrium. Changes in receptor levels, receptor desensitization, and locally produced oxytocin are factors that influence the effect of oxytocin on uterine contractility in labor. Activation of oxytocin receptor causes myometrial contractions by increasing intracellular Ca^+2^ and production of prostaglandins. Since oxytocin induces contractions, the inhibition of its action has been a target in the management of preterm labor. Atosiban is today the only oxytocin receptor antagonist that is available as a tocolytic. However, the quest for oxytocin receptor antagonists with a better pharmacological profile has led to the synthesis of peptide and nonpeptide molecules such as barusiban, retosiban, L-368,899, and SSR-126768A. Many of these oxytocin receptor antagonists are used only as pharmacological tools, while others have tocolytic action. In this paper, we summarize the action of oxytocin and its receptor and we present an overview of the clinical and experimental data of oxytocin antagonists and their tocolytic action.

## 1. Introduction

Oxytocin (OT) is a nonapeptide synthesized by the magnocellular neurons located in the supraoptic and paraventricular nuclei of the hypothalamus and secreted to the circulation by the posterior pituitary and nerve terminals in response to various stimuli. The sequence of amino acids in the OT molecule is Cysteine-Tyrosine-Isoleucine-Glutamine-Asparagine-Cysteine-Proline-Leucine-Glycinamide, with a sulfur bridge between the two cysteines. OT and vasopressin have similar structures and differ only in two amino acids. Oxytocin is also synthesized in many peripheral tissues, for example, uterus, placenta, amnion, corpus luteum, testis, and heart [1]. 

Oxytocin exerts a variety of actions and is involved in a large number of physiological and pathological processes. These actions include the regulation of the hypothalamo-pituitary-adrenal axis in response to stress, pregnancy, luteal function, maternal behavior, cell proliferation, modulation of emotional relationships and sexual behavior, erectile function and ejaculation, antinociception, cardiovascular function, osteoporosis, and neuropsychiatric disorders [2–6]. However, its best-known and most well-established roles are stimulation of uterine contractions during parturition and milk release during lactation. In 1906, Sir Henry Dale found that an extract from the human posterior pituitary gland had a uterotonic effect, and Vincent du Vigneaud et al. achieved synthesis of oxytocin in 1953 [7]. Since oxytocin contributes to myometrial contractility, its receptor has been a target for tocolytic agents. While atosiban is an oxytocin receptor (OTR) antagonist used for the management of preterm labor [8], research is ongoing for the tocolytic properties of various other OTR antagonists. 

## 2. The Oxytocin Receptor

**Figure 1:**
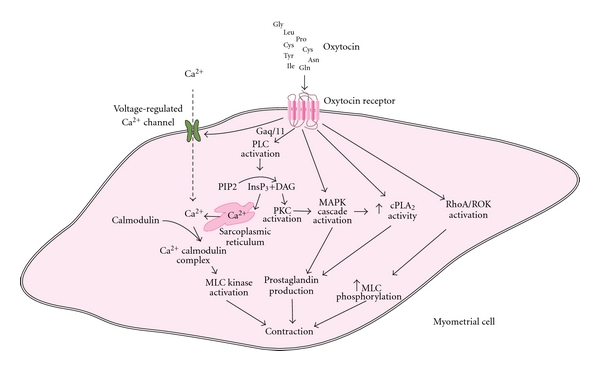
Oxytocin receptor linked signaling pathways resulting in myometrial contraction. Binding of OT to OTR activates G*α*_q/11_ and then PLC, which hydrolyses PIP2 to InsP3 and DAG. InsP3 causes release of Ca^2+^ from the sarcoplasmic reticulum, while DAG activates PKC. G*α*_q/11_ also causes activation of voltage-regulated Ca^2+^ channels and Ca^2+^ entry into the cells. Ca^2+^ binds to calmodulin, and the Ca^2+^-calmodulin complex activates MLC kinase, resulting in myometrial contraction. Both OTR and PKC activate the MAPK cascade, while OTR and MAPK result in increased cPLA2 activity. Increased cPLA2 activity and MAPK activation result in prostaglandin production, which also contributes to the contractile effect. The activation of the RhoA-ROK cascade by the OTR is another pathway that results in increased MLC phosphorylation and myometrial contraction. OT: oxytocin, OTR: oxytocin receptor, PLC: phospholipase, PIP2: phosphatidylinositol 4,5-bisphosphate, InsP3: inositol 1,4,5- triphosphate, DAG: diacylglycerol, PKC: protein kinases type C, MLC: myosin light-chain, MAPK: mitogen-activated protein kinase, cPLA2: cytosolic phospholipase A2, ROK: RhoA associated protein kinase.

The oxytocin receptor belongs to the rhodopsin-type class I G-protein-coupled receptor (GPCR) superfamily. The gene of the OTR contains 3 introns and 4 exons and is located in a single copy on chromosome 3p25. Apart from oxytocin, other molecules such as arginine vasopressin (AVP) and oxytocin agonists or antagonists are able, because of their similar structure, to bind to the receptor. Binding of OT to the cell surface transmembrane OTR activates the receptor which subsequently activates various intracellular signal pathways, this triggering the numerous effects of the hormone, including contraction (Figure 1). OTR is coupled to the G_q/11_ a-class guanosine triphosphate (GTP) binding proteins. The Gq/phospholipase C (PLC)/inositol 1,4,5-triphosphate (InsP3) pathway is the major pathway mediating the signal of OTR after binding of OT to its receptor. Binding of OT activates, through G*α*_q/11_, PLC, which hydrolyzes phosphatidylinositol 4,5-bisphosphate (PIP2) to InsP3 and diacylglycerol (DAG) [9]. InsP3 releases Ca^2+^ ions from intracellular stores, while DAG activates protein kinases type C (PKC), which further phosphorylates other proteins. Ca^2+^ ions bind to calmodulin, and the Ca^2+^-calmodulin complex activates the myosin light-chain kinase, thereby causing myometrial muscle contraction. The same mechanism additionally causes contraction of mammary myoepithelial cells and milk ejection [1]. However, OTR is also coupled with other G-proteins, Gs and Gi, which result in various cellular effects, among them the inhibition of cell growth [10]. 

OTR also acts on voltage-gated or receptor coupled channels, leading to membrane depolarization and the entry of extracellular Ca^2+^ to the cells, this further promoting smooth muscle contractility [11]. 

Moreover, OTR activates the mitogen-activated protein kinase (MAPK) and the Rho kinase pathways. Rho-associated protein kinases are implicated in many cellular phenomena, including cell contractility, cell migration, and cell cycle control [12]. Activations of OTR and MAPK both result in increased cytosolic phospholipase A2 (cPLA2) activity. cPLA2 hydrolyzes phospholipids and liberates arachidonic acid, which results in increased production of prostaglandins via cyclooxygenase-2 (COX-2), an enzyme that is upregulated by MAPK [13, 14]. The increased production of prostaglandins in conjunction with the increase in intracellular Ca^2+^ and the activation of the Rho and MAP kinase pathways results in the contractile effects of OT-OTR activation. 

## 3. The Oxytocin-Oxytocin Receptor in Labor

The mechanism of transition from myometrial quiescence to labor initiation is a complex one in which numerous diverse factors contribute in conjunction to the process. Hormones such as progesterone and CRH have a crucial role in this transition [15–17]. Immune responses, triggered via release of cytokines and other mediators of inflammation, are also important, especially in cases of preterm labor [18–22]. Oxytocin additionally contributes to the mechanism of labor and interacts with many of the other contributing factors. However, circulating oxytocin does not seem to be necessary for the initiation and completion of parturition in humans, since normal labor can be achieved in the absence of oxytocin, as in cases of pituitary dysfunction [23]. Furthermore, in humans oxytocin circulation levels do not increase significantly during pregnancy or the beginning of labor. However, some changes in circulating oxytocin occur as its levels are increased at the expulsive stage and pulsatile changes occur in pregnant women at term. As mentioned above, the oxytocin locally produced in the placenta is likely to be more important than circulating oxytocin for the mechanism of labor. Changes to the OTR also occur towards labor. OTR is upregulated at the end of gestation and in both term and preterm labor, and sensitivity to oxytocin-induced contractions is greatly increased [24–26]. Though it is assumed that steroid hormones may also influence the number of OTRs, the mechanisms of regulation are complex and not as yet fully understood [27–31]. It is of interest that in rabbits, myometrium under progesterone dominance is refractory to oxytocin [15].

Coordinated synchronous myometrial contractions during labor are achieved through the formation of gap junctions among cells, which are created by multimers of the protein connexin 43 [32, 33]. Oxytocin increases connexin-43 levels and upregulates morphologic gap junctions [34, 35]. After parturition, OTR expression in the mammary glands remains high thus facilitating lactation, while OT-binding sites in the uterus decline rapidly [36, 37].

Continuous exposure to high doses of oxytocin leads to desensitization of the receptor [38]. Desensitization is a phenomenon that is observed in GPCR receptors and occurs via different mechanisms such as phosphorylation, internalization, or changes at the receptor mRNA levels. Desensitization, whose purpose is to protect cells from overstimulation after prolonged agonist stimulation, can occur very rapidly, within seconds or minutes, and takes place in two steps. The first step is the phosphorylation of the receptor that inhibits G-protein activation, and the second step is the binding of proteins called arrestins, which prevent G-protein activation and promote receptor internalization. Evidence appears to indicate that receptors recycle back to the cell surface after internalization [39].

OTR desensitization is a phenomenon that occurs after prolonged agonist stimulation and may last for hours or even days [36]. The mRNA of the OTR is also reduced after continuous OT treatment. In cultured human myometrial cells, treatment with OT for up to 20 hours causes OTR desensitization and great reduction of OT-binding sites without receptor internalization. OTR mRNA levels are reduced, though not the total amount of OTR protein [40]. In vivo, when oxytocin is used to augment labor in pregnant women, there is also a reduction in myometrial oxytocin binding sites and in OTR mRNA levels [41]. Oxytocin receptor downregulation has great clinical significance, as prolonged oxytocin infusion may fail to augment labor or can lead to postpartum uterine atony that cannot be managed with additional oxytocin infusion. However, as oxytocin is normally secreted in pulses, this pulsatile secretion may prevent desensitization from occurring. This might explain why lower doses of oxytocin are required to augment labor when it is administered in pulses and higher doses when it is infused continuously [42].

## 4. Oxytocin Agonists

OTR agonist molecules have been developed and studied as pharmacological tools or as potential drugs for the management of neuropsychiatric diseases including anxiety-related disorders, schizophrenia, and autism. OTR agonists may be peptides ([Thr^4^]OT, [HO^1^][Thr^4^]OT, [Thr^4^,Gly^7^]OT, [HO^1^][Thr^4^,Gly^7^]OT and other molecules) or nonpeptides (WAY-267464 and other compounds) [43–45]. WAY-267464 exerts oxytocinergic actions, such as anxiolytic effects in mice [46]. 

Synthetic oxytocin is used to augment labor and treat postpartum hemorrhage. Demoxytocin is an oxytocin analogue that has been used for labor induction, though it has been proven less effective compared to prostaglandins [47]. Carbetocin is a peptide synthetic oxytocin analogue indicated for the prevention of uterine atony after cesarean section with spinal or epidural anesthesia: it has a longer half-life than oxytocin and possesses the advantage that it is administered in a single dose, intramuscularly or intravenously [48].

## 5. Oxytocin Antagonists as Tocolytic Agents

The therapeutic target in the treatment of preterm labor is currently the pharmacological inhibition of uterine contractions with the use of various tocolytic agents. Tocolytic agents are used to maintain pregnancy for 24–48 hours to allow corticosteroids administration to act and to permit the transfer of the mother to a center with a neonatal intensive care unit. Ritodrine (a beta-receptor agonist), nitric oxide donors (as glyceryl trinitrate), calcium-channel blockers, and COX-2 inhibitors such as indomethacin are some of the currently available tocolytics [8].

### 5.1. Peptide OTR Antagonists

**Figure 2:**
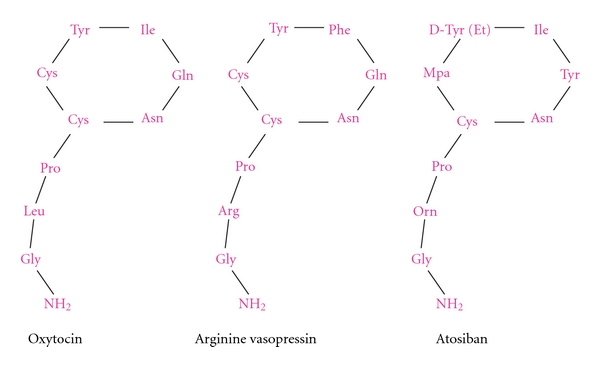
The structure of oxytocin, arginine vasopressin, and atosiban.

Selective human oxytocin receptors antagonists have also been synthesized as tocolytic agents for the management of preterm labor. Atosiban is an oxytocin analogue (1-Deamino-2-D-Tyr-(O-ethyl)-4-Thr-8-ornoxytocin) based on modification of some amino acids in the structure of oxytocin at positions 1, 2, 4, and 8 (Figure 2). It is a mixed vasopressin (V1a) and oxytocin receptor antagonist that blocks OT binding to OTR and is the only oxytocin antagonist used today for the treatment of preterm labor in Europe and other countries, though not in the USA. However, as it is also an antagonist of the vasopressin receptor V1a, this results in related undesirable effects. The onset of uterine relaxation after atosiban administration is rapid. Atosiban is given intravenously for up to 48 hours. Clinically, atosiban is as effective as *β*2-adrenergic agonists and with lesser adverse effects. When subcutaneously administered as maintenance therapy after a period of preterm labor, atosiban failed to reduce the incidence of preterm birth or improve neonatal outcome [49].

Clinically, atosiban is safer than beta-receptor agonists. Atosiban is comparable in clinical effectiveness to conventional beta-agonist therapy (ritodrine, salbutamol, or terbutaline), but it is associated with fewer maternal cardiovascular side effects and is better tolerated [50–53]. Compared to calcium channel blockers, atosiban has been shown equally effective as nifedipine and with fewer maternal side effects [54, 55]. Cyclooxygenase inhibitors also present significant side effects [56]. Conversely, there is at present insufficient evidence to recommend the use of nitric oxide donors as inhibitors of preterm delivery [57]. However, atosiban has not been proven to be superior in terms of neonatal outcome, as concerns have been described in some studies [58].

Unfortunately, atosiban displays some disadvantages when used as a tocolytic. It has limited bioavailability and requires parenteral administration and hospitalization, has low affinity of the OTR, and is also an antagonist for the V1a receptors that cause side effects. These limitations have led to efforts for the discovery of new peptide and nonpeptide OT antagonists for the management of preterm labor. Most of these substances are still being evaluated at an experimental level (Table 1), and clinical studies in most cases have not been successfully completed [43].

A number of highly selective OT peptidic antagonists have been designed and synthesized, like d(CH_2_)_5_[Tyr(Me)^2^]·OVT, desGly–NH_2_,d(CH_2_)_5_[Tyr(Me)^2^,Thr^4^]OVT, desGly–NH_2_,d(CH_2_)_5_[D-Tyr^2^,Thr^4^]OVT, d(CH_2_)^5^,[D-Thi^2^,Thr^4^,Tyr–NH_2_^9^]OVT, and desGly–NH_2_,d(CH_2_)_5_[D-Trp^2^,Thr^4^,Dap^5^]OVT. These molecules are both OTR and AVP receptor antagonists but are more potent as OT antagonists than as V1a antagonists. However, experiments have revealed striking differences between species concerning the affinity of many antagonists to the various receptors [59].

Some of the new peptide OT/AVP antagonists have higher affinity for human receptor than the peptide atosiban. These new peptides are desGly–NH_2_,d(CH_2_)_5_[D-2-Nal^2^,Thr^4^]OVT, desGly–NH_2_,d(CH_2_)_5_[2-Nal^2^,Thr^4^]OVT, d(CH_2_)_5_[D-2-Nal^2^,Thr^4^,Tyr–NH_2_^9^]OVT, and d(CH_2_)_5_[2-Nal^2^,Thr^4^,Tyr–NH_2_^9^]OVT and may be candidates as potential tocolytic agents [43].

Barusiban is a selective peptide oxytocin antagonist with a high affinity for the human OTR and low for the V1a receptor. It has a higher potency and a longer duration of action than atosiban. In contractility studies with isolated human myometrium, barusiban inhibits oxytocin-induced myometrial contractions of both preterm and term myometrium, and this action was at least as potent as the action of atosiban [60]. In nonhuman primates, barusiban also inhibits oxytocin-induced myometrial contractions [61, 62]. In pregnant monkeys, in which atosiban and barusiban were tested following induction of contractions by OT, the duration of action of barusiban was generally longer than that of atosiban (13–15 hours compared to 1.5–3 hours). For long-term treatment, continuous high-dose infusions of barusiban or the beta-2 agonist fenoterol were administered: barusiban moderated intrauterine pressure increase in response to daily OT challenge and prolonged pregnancy more effectively than fenoterol [63].

 However, despite the promising results in nonhuman primates, in a recent study in pregnant women, barusiban was not more effective than placebo in stopping preterm labor. In a multicenter trial that was conducted at 21 participating centers in 6 different European countries, participants were randomly assigned to receive a single intravenous bolus dose of placebo or barusiban. There was no significant difference between the placebo group and any of the barusiban groups with regard to the percentage of women who did not deliver within 48 hours, nor was there any significant decrease in the number of uterine contractions between the barusiban groups and placebo group. Additionally, there were no statistical differences in maternal or neonatal adverse effects between placebo and barusiban groups [64].

### 5.2. Nonpeptide OTR Antagonists

Since peptide antagonists lack oral bioavailability, pharmaceutical companies have searched for an effective nonpeptide oxytocin antagonist. GSK221149A (2′-methyl-1′,3′-oxazol-4′-yl morpholine amide derivative 74), known as retosiban, is such a nonpeptide oxytocin antagonist. In vitro experiments using Chinese hamster ovary (CHO) cell membranes expressing human OT receptors or human vasopressin (V1a, V1b, V2) receptors and human endothelial kidney (HEK) cells expressing rat oxytocin receptors revealed that retosiban has a higher affinity for human and rat oxytocin receptors than for V1a and V2 receptors. In rats, when administered orally or intravenously, retosiban produced a dose-dependent decrease in oxytocin-induced uterine contraction. This effect was observed after either single or multiple dosing for 4 days. Also, spontaneous uterine contractions in late-term pregnant rats (at 19–21 days gestation) were significantly reduced by intravenous administration of retosiban [65]. Retosiban is more than 15-fold more potent compared to atosiban for the OTR [66]: it is at present on a Phase ll Clinical trial investigating its action as a tocolytic, but the results have not as yet been published [67].

Another nonpeptide oxytocin antagonist is L-368,899 (1-(((7,7-Dimethyl-2(S)-(2(S)-amino-4-(methylsulfonyl) butyramido) bicyclo [2.2.1]-heptan-1(S)-yl)methyl) sulfonyl)-4(2methylphenyl) piperazine). In pregnant rhesus monkeys, L-368,899 was shown to be a potent OT antagonist that inhibits spontaneous nocturnal uterine contractions. In women, L-368,899 also blocked OT-stimulated uterine activity postpartum with a potency similar to that observed in the pregnant rhesus monkey [68]. However, no further clinical evaluation was undertaken because of suboptimal oral bioavailability and pharmacokinetics [69]. L-368,899 is also brain penetrant, and experiments in one adult female monkey demonstrated that it altered maternal and sexual behavior [4].

SSR-126768A (4-Chloro-3-[(3R)-(+)-5-chloro-1-(2,4-dimethoxybenzyl)-3-methyl-2-oxo-2,3-dihydro-1H-indol-3-yl]-N-ethyl-N-(3-pyridylmethyl)-benzamide, Hydrochloride), a nonpeptide oxytocin antagonist, produced a competitive antagonistic effect against OT in rat myometrial strips, as in conscious rat after oral administration. After oral administration in pregnant rats in labor, SSR-126768A significantly delayed parturition, similarly to ritodrine, and had a rapid onset of action, and its duration was still observed 24 hours after treatment. Experimentally, SSR-126768A inhibited the response to OT at-term human pregnant uterus sections, this effect being observed in a concentration-dependent manner [70].

As nonpeptide OTR antagonists display high selectivity for the OTR receptor, they may provide orally administered tocolytics for both the acute and long-term management of preterm labor, which would undoubtedly offer a significant improvement in neonatal outcomes.

In women who go into preterm labor, relcovaptan (which is a nonpeptide vasopressin V1a receptor antagonist) was reported to inhibit uterine contractions [71, 72]. In a study in 18 women in preterm labor between 32–36 weeks, 12 patients received at random a single oral dose of relcovaptan and 6 patients received placebo, while uterine contractions, were monitored up to 6 h after administration. Relcovaptan inhibited uterine contractions, and, compared with placebo, the difference was statistically significant. Relcovaptan has also revealed positive initial results in tests against Raynaud's disease, dysmenorrhoea, and experimentally in ischemic brain damage, although it has not yet been approved for clinical use [73–76].

## 6. Conclusion

Oxytocin is a neuropeptide which affects uterine contractility during labor. Its receptor is a transmembrane receptor belonging to the G-protein-coupled receptor superfamily. The main signaling pathway is the Gq/LPC/Ins3 pathway, but the MAPK and the RhoA/Rho kinase pathways are also activated, contributing to increased prostaglandin production and direct contractile effect on myometrial cells. Various peptide and nonpeptide antagonists have been developed as potential tocolytic agents or research tools for the various OT functions. Atosiban is currently available for use as a tocolytic agent. However, other oxytocin antagonists, both peptide and nonpeptide, such as barusiban, retosiban, L-368,899, and SSR-126768, have displayed tocolytic action and are under further investigation. The future use of OTR antagonists possessing a better pharmacological profile than atosiban is a promising research field that aims to further improve the management of preterm labor.

## Figures and Tables

**Table 1:** The most extensively studied OT antagonists and their experimental or clinical applications.

Oxytocin antagonists	Organism	Experimental or therapeutic application
(I)* Peptide *		

1-Deamino-2-D-Tyr-(O-ethyl)-4-Thr-8-ornoxytocin (Atosiban)	Human	Clinically used tocolytic agent
d(CH_2_)_5_[Tyr(Me)^2^]OVT	Animals/human cells and tissues, or both	Pharmacological tool in experimental studies of OT functions
desGly–NH_2_,d(CH_2_)_5_[Tyr(Me)^2^,Thr^4^]OVT	Animals/human cells and tissues, or both	Pharmacological tool in experimental studies of OT functions
desGly–NH_2_,d(CH_2_)_5_[D-Tyr^2^,Thr^4^]OVT	Animals/human cells and tissues, or both	Pharmacological tool in experimental studies of OT functions
d(CH_2_)^5^,[D-Thi^2^,Thr^4^,Tyr–NH_2_^9^]OVT	Animals/human cells and tissues, or both	Pharmacological tool in experimental studies of OT functions
desGly–NH_2_,d(CH_2_)_5_[D-Trp^2^,Thr^4^,Dap^5^]OVT	Animals/human cells and tissues, or both	Pharmacological tool in experimental studies of OT functions
desGly–NH_2_,d(CH_2_)_5_[D-2-Nal^2^,Thr^4^]OVT	Animals/human cells and tissues, or both	Pharmacological tool in experimental studies of OT functions
desGly–NH_2_,d(CH_2_)_5_[2-Nal^2^,Thr^4^]OVT	Animals/human cells and tissues, or both	Pharmacological tool in experimental studies of OT functions
d(CH_2_)_5_[D-2-Nal^2^,Thr^4^,Tyr–NH_2_^9^]OVT	Animals/human cells and tissues, or both	Pharmacological tool in experimental studies of OT functions
d(CH_2_)_5_[2-Nal^2^,Thr^4^,Tyr–NH_2_^9^]OVT	Animals/human cells and tissues, or both	Pharmacological tool in experimental studies of OT functions
FE 200 400 (Barusiban)	Monkeys/human myometrial tissue	Pharmacological tool in experimental studies of OT functions

(II)* Nonpeptide *		

GSK221149A (Retosiban)	Rats	Tocolytic action. A phase ll clinical trial for its use as a tocolytic in humans has not yet been completed
L-368,899	Animals/human cells and tissues, or both	Pharmacological tools in experimental studies of OT function. CNS effects. Inhibits spontaneous nocturnal uterine contractions in pregnant rhesus monkeys. Clinical studies discontinued
L-371,257	Animals/human cells and tissues, or both	Pharmacological tool in experimental studies of OT functions
WAY-162720	Animals/human cells and tissues, or both	Pharmacological tool in experimental studies of OT functions
SSR-126768A	Rats/human myometrial tissue	Tocolytic action in rats. Inhibits the response to OT in term human pregnant uterine sections

## References

[1] Gimpl G, Fahrenholz F (2001). The oxytocin receptor system: structure, function, and regulation. *Physiological Reviews*.

[2] Viero C, Shibuya I, Kitamura N (2010). Oxytocin: crossing the bridge between basic science and pharmacotherapy. *CNS Neuroscience and Therapeutics*.

[3] Lee HJ, Macbeth AH, Pagani JH, Scott Young W (2009). Oxytocin: the great facilitator of life. *Progress in Neurobiology*.

[4] Boccia ML, Goursaud APS, Bachevalier J, Anderson KD, Pedersen CA (2007). Peripherally administered non-peptide oxytocin antagonist, L368,899®, accumulates in limbic brain areas: a new pharmacological tool for the study of social motivation in non-human primates. *Hormones and Behavior*.

[5] Yang J (1994). Intrathecal administration of oxytocin induces analgesia in low back pain involving the endogenous opiate peptide system. *Spine*.

[6] Schaller F, Watrin F, Sturny R, Massacrier A, Szepetowski P, Muscatelli F (2010). A single postnatal injection of oxytocin rescues the lethal feeding behaviour in mouse newborns deficient for the imprinted Magel2 gene. *Human Molecular Genetics*.

[7] du Vigneaud V, Ressler C, Swan JM, Roberts CW, Katsoyannis PG (1954). The synthesis of oxytocin. *Journal of the American Chemical Society*.

[8] Simhan HN, Caritis SN (2007). Prevention of preterm delivery. *The New England Journal of Medicine*.

[9] Smith R (2007). Parturition. *The New England Journal of Medicine*.

[10] Rimoldi V, Reversi A, Taverna E (2003). Oxytocin receptor elicits different EGFR/MAPK activation patterns depending on its localization in caveolin-1 enriched domains. *Oncogene*.

[11] Sanborn BM, Ku CY, Shlykov S, Babich L (2005). Molecular signaling through G-protein-coupled receptors and the control of intracellular calcium in myometrium. *Journal of the Society for Gynecologic Investigation*.

[12] Riento K, Ridley AJ (2003). Rocks: multifunctional kinases in cell behaviour. *Nature Reviews Molecular Cell Biology*.

[13] Molnar M, Hertelendy F (1995). Signal transduction in rat myometrial cells: comparison of the actions of endothelin-1, oxytocin and prostaglandin F(2*α*). *European Journal of Endocrinology*.

[14] Soloff MS, Jeng YJ, Copland JA, Strakova Z, Hoare S (2000). Signal pathways mediating oxytocin stimulation of prostaglandin synthesis in select target cells. *Experimental Physiology*.

[15] Csapo A (1956). Progesterone “block”. *American Journal of Anatomy*.

[16] Mesiano S, Wang Y, Norwitz ER (2011). Progesterone receptors in the human pregnancy uterus: do they hold the key to birth timing?. *Reproductive Sciences*.

[17] Mclean M, Bisits A, Davies J, Woods R, Lowry P, Smith R (1995). A placental clock controlling the length of human pregnancy. *Nature Medicine*.

[18] Christiaens I, Zaragoza DB, Guilbert L, Robertson SA, Mitchell BF, Olson DM (2008). Inflammatory processes in preterm and term parturition. *Journal of Reproductive Immunology*.

[19] Malamitsi-Puchner A, Vrachnis N, Samoli E, Baka S, Hassiakos D, Creatsas G (2006). Elevated second trimester amniotic fluid interferon *γ*-inducible T-cell *α* chemoattractant concentrations as a possible predictor of preterm birth. *Journal of the Society for Gynecologic Investigation*.

[20] Malamitsi-Puchner A, Vrachnis N, Samoli E (2006). Investigation of midtrimester amniotic fluid factors as potential predictors of term and preterm deliveries. *Mediators of Inflammation*.

[21] Vrachnis N, Vitoratos N, Iliodromiti Z, Sifakis S, Deligeoroglou E, Creatsas G (2010). Intrauterine inflammation and preterm delivery. *Annals of the New York Academy of Sciences*.

[22] Mittal P, Romero R, Tarca AL (2010). Characterization of the myometrial transcriptome and biological pathways of spontaneous human labor at term. *Journal of Perinatal Medicine*.

[23] Phelan JP, Guay AT, Newman C (1978). Diabetes insipidus in pregnancy: a case review. *American Journal of Obstetrics and Gynecology*.

[24] Fuchs AR, Fuchs F, Husslein P (1982). Oxytocin receptors and human parturition: a dual role for oxytocin in the initiation of labor. *Science*.

[25] Fuchs AR, Fuchs F, Husslein P, Soloff MS (1984). Oxytocin receptors in the human uterus during pregnancy and parturition. *American Journal of Obstetrics and Gynecology*.

[26] Petraglia F, Florio P, Nappi C, Genazzani AR (1996). Peptide signaling in human placenta and membranes: autocrine, paracrine, and endocrine mechanisms. *Endocrine Reviews*.

[27] Horn S, Bathgate R, Lioutas C, Bracken K, Ivell R (1998). Bovine endometrial epithelial cells as a model system to study oxytocin receptor regulation. *Human Reproduction Update*.

[28] Mirando MA, Ott TL, Vallet JL, Davis M, Bazer FW (1990). Oxytocin-stimulated inositol phosphate turnover in endometrium of ewes is influenced by stage of the estrous cycle, pregnancy, and intrauterine infusion of ovine conceptus secretory proteins. *Biology of Reproduction*.

[29] Vallet JL, Lamming GE, Batten M (1990). Control of endometrial oxytocin receptor and uterine response to oxytocin by progesterone and oestradiol in the ewe. *Journal of Reproduction and Fertility*.

[30] Wathes DC, Mann GE, Payne JH, Riley PR, Stevenson KR, Lamming GE (1996). Regulation of oxytocin, oestradiol and progesterone receptor concentrations in different uterine regions by oestradiol, progesterone and oxytocin in ovariectomized ewes. *Journal of Endocrinology*.

[31] Zingg HH, Rozen F, Breton C (1996). Gonadal steroid regulation of oxytocin and oxytocin receptor gene expression. *Advances in Experimental Medicine and Biology*.

[32] Garfield RE, Sims S, Daniel EE (1977). Gap junctions: their presence and necessity in myometrium during parturition. *Science*.

[33] Garfield RE, Hayashi RH (1981). Appearance of gap junctions in the myometrium of women during labor. *American Journal of Obstetrics and Gynecology*.

[34] Ambrus G, Rao CV (1994). Novel regulation of pregnant human myometrial smooth muscle cell gap junctions by human chorionic gonadotropin. *Endocrinology*.

[35] Khan-Dawood FS, Yang J, Dawood MY (1998). Hormonal regulation of connexin-43 in baboon corpora lutea. *Journal of Endocrinology*.

[36] Terzidou V (2007). Biochemical and endocrinological preparation for parturition. *Best Practice and Research: Clinical Obstetrics and Gynaecology*.

[37] Petraglia F, Imperatore A, Challis JRG (2010). Neuroendocrine mechanisms in pregnancy and parturition. *Endocrine Reviews*.

[38] Plested CP, Bernal AL (2001). Desensitisation of the oxytocin receptor and other G-protein coupled receptors in the human myometrium. *Experimental Physiology*.

[39] Conti F, Sertic S, Reversi A, Chini B (2009). Intracellular trafficking of the human oxytocin receptor: evidence of receptor recycling via a Rab4/Rab5 “short cycle”. *American Journal of Physiology*.

[40] Phaneuf S, Asbóth G, Carrasco MP (1998). Desensitization of oxytocin receptors in human myometrium. *Human Reproduction Update*.

[41] Phaneuf S, Rodriguez Linares B, Tamby Raja RL, MacKenzie IZ, Lopez Bernal A (2000). Loss of myometrial oxytocin receptors during oxytocin-induced and oxytocin-augmented labour. *Journal of Reproduction and Fertility*.

[42] Dawood MY (1996). Novel approach to oxytocin induction-augmentation of labor: application of oxytocin physiology during pregnancy. *Advances in Experimental Medicine and Biology*.

[43] Manning M, Stoev S, Chini B, Durroux T, Mouillac B, Guillon G (2008). Peptide and non-peptide agonists and antagonists for the vasopressin and oxytocin V1a, V1b, V2 and OT receptors: research tools and potential therapeutic agents. *Progress in Brain Research*.

[44] Pitt G, Batt A, Haigh R (2004). Non-peptide oxytocin agonists. *Bioorganic and Medicinal Chemistry Letters*.

[45] Borthwick AD (2006). Oxytocin antagonists and agonists. *Annual Reports in Medicinal Chemistry*.

[46] Ring RH, Schechter LE, Leonard SK (2010). Receptor and behavioral pharmacology of WAY-267464, a non-peptide oxytocin receptor agonist. *Neuropharmacology*.

[47] Westergaard JG, Lange AP, Pedersen GT, Secher NJ (1983). Use of oral oxytocics for stimulation of labor in cases of premature rupture of the membranes at term. A randomized comparative study of prostaglandin E2 tablets and demoxytocin resoriblets. *Acta Obstetricia et Gynecologica Scandinavica*.

[48] Rath W (2009). Prevention of postpartum haemorrhage with the oxytocin analogue carbetocin. *European Journal of Obstetrics Gynecology and Reproductive Biology*.

[49] Papatsonis D, Flenady V, Liley H (2009). Maintenance therapy with oxytocin antagonists for inhibiting preterm birth after threatened preterm labour. *Cochrane Database of Systematic Reviews*.

[50] Bittar RE, Zugaib M (2009). Management of preterm labor. *Revista Brasileira de Ginecologia e Obstetricia*.

[51] Worldwide Atosiban versus Beta-agonists Study Group (2001). Effectiveness and safety of the oxytocin antagonist atosiban versus beta-adrenergic agonists in the treatment of preterm labour. he Worldwide Atosiban versus Beta-agonists Study Group. *British Journal of Obstetrics and Gynaecology*.

[52] Goodwin TM, Valenzuela GJ, Silver H, Creasy G (1996). Dose ranging study of the oxytocin antagonist atosiban in the treatment of preterm labor. Atosiban Study Group. *Obstetrics and Gynecology*.

[53] Moutquin JM, Sherman D, Cohen H (2000). Double-blind, randomized, controlled trial of atosiban and ritodrine in the treatment of preterm labor: a multicenter effectiveness and safety study. *American Journal of Obstetrics and Gynecology*.

[54] Kashanian M, Akbarian AR, Soltanzadeh M (2005). Atosiban and nifedipin for the treatment of preterm labor. *International Journal of Gynecology and Obstetrics*.

[55] Al-Omari WR, Al-Shammaa HB, Al-Tikriti EM, Ahmed KW (2006). Atosiban and nifedipine in acute tocolysis: a comparative study. *European Journal of Obstetrics Gynecology and Reproductive Biology*.

[56] King J, Flenady V, Cole S, Thornton S (2005). Cyclo-oxygenase (COX) inhibitors for treating preterm labour. *Cochrane Database of Systematic Reviews*.

[57] Duckitt K, Thornton S (2002). Nitric oxide donors for the treatment of preterm labour. *Cochrane Database of Systematic Reviews*.

[58] Papatsonis D, Flenady V, Cole S, Liley H (2005). Oxytocin receptor antagonists for inhibiting preterm labour. *Cochrane Database of Systematic Reviews*.

[59] Chini B, Manning M, Guillon G (2008). Affinity and efficacy of selective agonists and antagonists for vasopressin and oxytocin receptors: an "easy guide" to receptor pharmacology. *Progress in Brain Research*.

[60] Pierzynski P, Lemancewicz A, Reinheimer T, Akerlund M, Laudanski T (2004). Inhibitory effect of barusiban and atosiban on oxytocin-induced contractions of myometrium from preterm and term pregnant women. *Journal of the Society for Gynecologic Investigation*.

[61] Reinheimer TM, Chellman GJ, Resendez JC, Meyer JK, Bee WH (2006). Barusiban, an effective long-term treatment of oxytocin-induced preterm labor in nonhuman primates. *Biology of Reproduction*.

[62] Reinheimer TM, Bee WH, Resendez JC, Meyer JK, Haluska GJ, Chellman GJ (2005). Barusiban, a new highly potent and long-acting oxytocin antagonist: pharmacokinetic and pharmacodynamic comparison with atosiban in a cynomolgus monkey model of preterm labor. *Journal of Clinical Endocrinology and Metabolism*.

[63] Reinheimer TM (2007). Barusiban suppresses oxytocin-induced preterm labour in non-human primates. *BMC Pregnancy and Childbirth*.

[64] Thornton S, Goodwin TM, Greisen G, Hedegaard M, Arce JC (2009). The effect of barusiban, a selective oxytocin antagonist, in threatened preterm labor at late gestational age: a randomized, double-blind, placebo-controlled trial. *American Journal of Obstetrics and Gynecology*.

[65] McCafferty GP, Pullen MA, Wu C (2007). Use of a novel and highly selective oxytocin receptor antagonist to characterize uterine contractions in the rat. *American Journal of Physiology*.

[66] Borthwick AD, Liddle J (2011). The design of orally bioavailable 2,5-diketopiperazine oxytocin antagonists: from concept to clinical candidate for premature labor. *Medicinal Research Reviews*.

[67] http://clinicaltrials.gov/ct2/show/study/NCT00404768.

[68] Pettibone DJ, Guidotti M, Harrell CM (1996). Progress in the development of oxytocin antagonists for use in preterm labor. *Advances in Experimental Medicine and Biology*.

[69] Freidinger RM, Pettibone DJ (1997). Small molecule ligands for oxytocin and vasopressin receptors. *Medicinal Research Reviews*.

[70] Serradeil-Le Gal C, Valette G, Foulon L (2004). SSR126768A (4-Chloro-3-[(3R)-(+)-5-chloro-1-(2,4-dimethoxybenzyl)-3-methyl-2-oxo-2, 3-dihydro-1H-indol-3-yl]-N-ethyl-N-(3-pyridylmethyl)-benzamide, Hydrochloride): a new selective and orally active oxytocin receptor antagonist for the prevention of Preterm labor. *Journal of Pharmacology and Experimental Therapeutics*.

[71] Åkerlund M, Bossmar T, Brouard R (1999). Receptor binding of oxytocin and vasopressin antagonists and inhibitory effects on isolated myometrium from preterm and term pregnant women. *British Journal of Obstetrics and Gynaecology*.

[72] Steinwall M, Bossmar T, Brouard R (2005). The effect of relcovaptan (SR 49059), an orally active vasopressin V 1a receptor antagonist, on uterine contractions in preterm labor. *Gynecological Endocrinology*.

[73] Decaux G, Soupart A, Vassart G (2008). Non-peptide arginine-vasopressin antagonists: the vaptans. *The Lancet*.

[74] Hayoz D, Bizzini G, Noël B (2000). Effect of SR 49059, a V1a vasopressin receptor antagonist, in Raynaud’s phenomenon. *Rheumatology*.

[75] Brouard R, Bossmar T, Fournié-Lloret D, Chassard D, Åkerlund M (2000). Effect of SR49059, an orally active V(1a) vasopressin receptor antagonist, in the prevention of dysmenorrhoea. *British Journal of Obstetrics and Gynaecology*.

[76] Shuaib A, Wang CX, Yang T, Noor R (2002). Effects of nonpeptide V1 vasopressin receptor antagonist SR-49059 on infarction volume and recovery of function in a focal embolic stroke model. *Stroke*.

